# Recognition of the polycistronic nature of human genes is critical to understanding the genotype-phenotype relationship

**DOI:** 10.1101/gr.230938.117

**Published:** 2018-05

**Authors:** Marie A. Brunet, Sébastien A. Levesque, Darel J. Hunting, Alan A. Cohen, Xavier Roucou

**Affiliations:** 1 Biochemistry Department, Université de Sherbrooke, Quebec J1E 4K8, Canada;; 2 Groupe de recherche PRIMUS, Department of Family and Emergency Medicine, Quebec J1H 5N4, Canada;; 3 PROTEO, Quebec Network for Research on Protein Function, Structure, and Engineering, Université Laval, Quebec G1V 0A6, Canada;; 4 Pediatric Department, Centre Hospitalier de l’Université de Sherbrooke, Quebec J1H 5N4, Canada;; 5 Department of Nuclear Medicine & Radiobiology, Université de Sherbrooke, Quebec J1H 5N4, Canada

## Abstract

Technological advances promise unprecedented opportunities for whole exome sequencing and proteomic analyses of populations. Currently, data from genome and exome sequencing or proteomic studies are searched against reference genome annotations. This provides the foundation for research and clinical screening for genetic causes of pathologies. However, current genome annotations substantially underestimate the proteomic information encoded within a gene. Numerous studies have now demonstrated the expression and function of alternative (mainly small, sometimes overlapping) ORFs within mature gene transcripts. This has important consequences for the correlation of phenotypes and genotypes. Most alternative ORFs are not yet annotated because of a lack of evidence, and this absence from databases precludes their detection by standard proteomic methods, such as mass spectrometry. Here, we demonstrate how current approaches tend to overlook alternative ORFs, hindering the discovery of new genetic drivers and fundamental research. We discuss available tools and techniques to improve identification of proteins from alternative ORFs and finally suggest a novel annotation system to permit a more complete representation of the transcriptomic and proteomic information contained within a gene. Given the crucial challenge of distinguishing functional ORFs from random ones, the suggested pipeline emphasizes both experimental data and conservation signatures. The addition of alternative ORFs in databases will render identification less serendipitous and advance the pace of research and genomic knowledge. This review highlights the urgent medical and research need to incorporate alternative ORFs in current genome annotations and thus permit their inclusion in hypotheses and models, which relate phenotypes and genotypes.

## The now irrefutable existence of “alternative” proteins

Recent work has revealed that genomes harbor many nonannotated open reading frames (ORFs) (Vanderperre et al. 2011; Bergeron et al. 2013; Anderson et al. 2015; Mouilleron et al. 2016; D'Lima et al. 2017; Plaza et al. 2017). Although two decades have passed since the first eukaryotic genome was sequenced, assigning translated ORFs to genetic loci remains a daunting task (Basrai et al. 1997; Claverie et al. 1997; Ladoukakis et al. 2011). Indeed, current genome annotations rely partly on ORF prediction algorithms that are only reliable for sequences beyond a certain length. Consequently, three main criteria are implemented to distinguish “true” ORFs from random events: the use of an ATG start codon, a minimum length of 100 codons, and a limit of a single ORF per transcript (Cheng et al. 2011; Andrews and Rothnagel 2014; Saghatelian and Couso 2015; Plaza et al. 2017). These criteria result in an important underestimation of translated ORFs in the genome (Andrews and Rothnagel 2014; Saghatelian and Couso 2015; Couso and Patraquim 2017; Plaza et al. 2017). With functional evidence for previously unannotated ORFs in bacteria (Wadler and Vanderpool 2007; Hemm et al. 2008, 2010; Storz et al. 2014; Lluch-Senar et al. 2015; Baek et al. 2017), *Drosophila* (Galindo et al. 2007; Kondo et al. 2007; Reinhardt et al. 2013; Aspden et al. 2014; Albuquerque et al. 2015; Li et al. 2016a; Pueyo et al. 2016a), plants (Hanada et al. 2013; Juntawong et al. 2014; Hsu et al. 2016; Hsu and Benfey 2017), and other eukaryotes (Oyama et al. 2007; Ingolia et al. 2011; Vanderperre et al. 2013; Ma et al. 2014), genome annotations will need to be revised.

**Figure 1.**
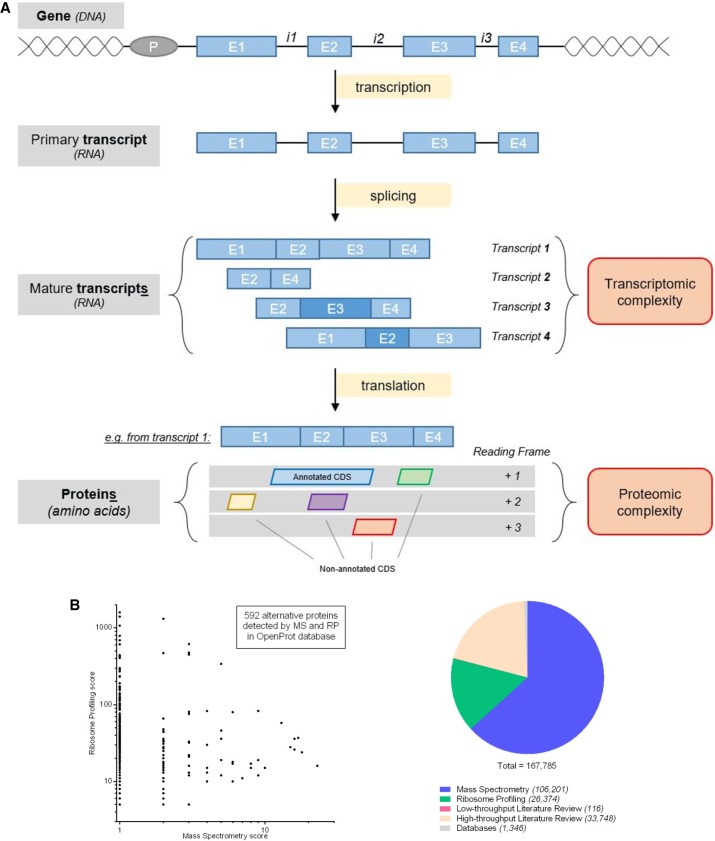
Schema of the transcriptomic and proteomic complexity inherent to a gene. (*A*) Genomic complexity representation. A gene is represented with a promoter (P) and introns (i) and exons (E). Splicing events lead to a suite of transcripts with frameshifted exons (darker blue shade), skipped exons, or retained introns. Then, proteomic complexity comes from each transcript with ORFs from any reading frames. However, now only one CDS is annotated per transcript, leaving an entire hidden proteome (unannotated CDSs). (*B*) Alternative ORFs databases. The OpenProt database predicts every ORF longer than 30 codons and reports experimental detection evidence for each of them. Five hundred ninety-two alternative ORFs were detected by both ribosome profiling (RP) and mass spectrometry (MS). The SmProt database reports smORFs (<100 codons) in different data sets (mass spectrometry, ribosome profiling, literature mining, and databases).

These “hidden” ORFs are found in multiple places within RNA: within long noncoding RNAs (lncRNAs), within 5′ and 3′ “untranslated” regions (UTRs) of mRNAs, or overlapping canonical coding sequences (CDSs) in an alternative reading frame (Slavoff et al. 2013; Mouilleron et al. 2016). They are, in general, notably smaller than annotated CDSs, but they are not limited to small ORFs (smORFs—ORFs smaller than 100 codons) (Samandi et al. 2017). Here, we define alternative ORFs as any coding sequence with an ATG start codon encoded within any reading frame of either lncRNAs or known coding mRNAs (either in UTRs or overlapping the CDS). Such a definition of ORFs allows for a more exhaustive yet more complex view of the genomic landscape. As shown in Figure 1A, a gene inherently carries transcriptomic complexity (RNA splicing events leading to multiple transcripts and thus to a suite of isoforms) and proteomic complexity (more than one protein per transcript). Even though the transcriptomic complexity is now widely accepted, consideration of proteomic complexity is usually restricted to one protein (and its splicing-derived isoforms) per gene. Protein complexity can arise from multiple sources, such as RNA splicing and editing, post-translational modifications, alternative initiation (internal ribosome entry site), stop codon read-through, or non-AUG initiation (Dunn et al. 2013; Venne et al. 2014; Ingolia 2016; Nishikura 2016; Blencowe 2017; Li et al. 2018). Notwithstanding, this review will focus on the proteomic complexity resulting from proteins encoded in alternative ORFs.

Recently, the development of new techniques or the optimization of existing ones has allowed for large-scale detection of alternative proteins and a more in-depth view of a cell proteomic landscape (Boekhorst et al. 2011; Aspden et al. 2014; Calviello et al. 2016; Hellens et al. 2016; Ma et al. 2016; Pueyo et al. 2016a; Delcourt et al. 2017; Hsu and Benfey 2017; Willems et al. 2017). Three detection methods—ribosome profiling, proteogenomics, and conservation signatures—have been used to identify likely translated and functional alternative ORFs, as further discussed later in this review. Such experimental data have been compiled in the sORFs repository, SmProt, and OpenProt databases (Olexiouk et al. 2016; Hao et al. 2017; openprot.org). The SmProt database reports 167,785 small proteins (mostly from lncRNAs) in the human genome, including 106,201 identified via mass spectrometry data and 26,374 via ribosome profiling (Fig. 1B; Hao et al. 2017). Comparatively, the OpenProt database currently reports 28,007 alternative proteins with experimental evidence, including 20,919 detected by mass spectrometry and 7680 by ribosome profiling; 592 alternative proteins were detected in both mass spectrometry and ribosome profiling experiments (Fig. 1B; openprot.org).

The number of reported alternative proteins varies between the databases, as they uphold different definitions of alternative ORFs (start codon other than ATG, length threshold, number of studies analyzed, and pipeline stringency). Indeed, the start codon use (restricted to ATG or not) and length threshold (<100 codons, or >30 codons) implemented will significantly alter the number of predicted alternative proteins. Subsequently, this will affect the sensitivity and specificity of detection methods, especially if the mass spectrometry identification pipeline is not adapted to an increase in the search space (Guthals et al. 2015). The various databases enforce different identification pipelines and stringencies on re-analysis of published data sets. This would inevitably lead to discrepancies in numbers of detected alternative ORFs. Here, we advocate for a cautious interpretation of the data, encouraging identifications made with different techniques and across several studies (identifications from mass spectrometry and ribosome profiling) (Fig. 1B).

These detection methods—ribosome profiling, proteogenomics, and conservation signatures—are revealing this hidden proteome, a novel repertoire for biomarkers and therapeutic strategies (Couso and Patraquim 2017; Karginov et al. 2017; Plaza et al. 2017). Here, we briefly review functional evidence for the biological roles of alternative proteins.

### SmORFs: mRNA or lncRNA ORFs smaller than 100 codons

**Figure 2.**
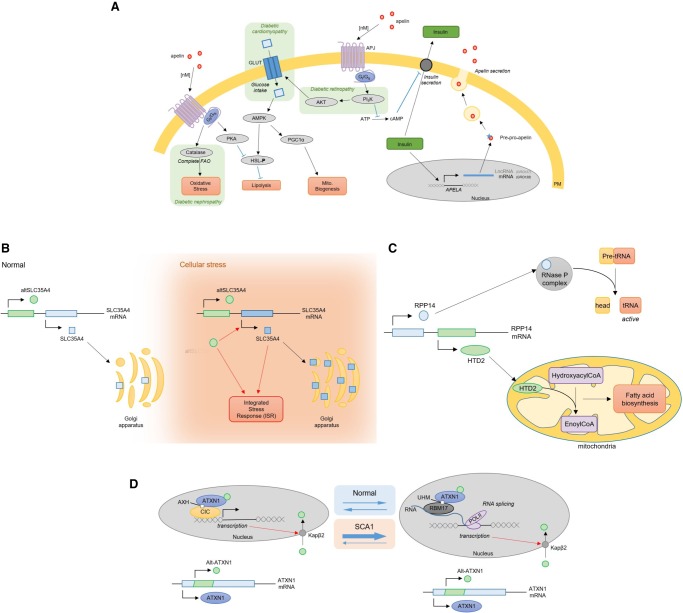
Examples of biologically important alternative ORFs. (*A*) Apelin, from overlooked to metabolic regulator. Apelin is encoded in an mRNA (GRCh38), previously annotated lncRNA (GRCh37), and subsequently secreted. Upon binding with APLNR (also known as APJ) receptor at a nanomolar range, it stimulates different metabolic pathways (glucose uptake, fatty acid oxidation, and mitochondrial biogenesis) and inhibits others (lipolysis and insulin secretion). These pathways are also involved in diabetic complications (cardiomyopathy, nephropathy, and retinopathy). Blue arrows represent inhibitory relationships, pathways involved in diabetic complications are highlighted in green. FAO: fatty acid oxidation; PM: plasma membrane; lncRNA: long non-coding RNA; Mito: mitochondrial. (*B*) SLC35A4 and its uORF-encoded protein, alt-SLC35A4. The SLC35A4 mRNA encodes two ORFs. Under physiological conditions, the canonical ORF, SLC35A4, is weakly expressed. The uORF-encoded protein alt-SLC35A4 is suspected to be the major protein product. Under cellular stress, both proteins are expressed. The alt-SLC35A4 expression level remains unchanged but positively regulates expression of SLC35A4. Both proteins are thought to be involved in the integrated stress response. ISR: integrated stress response. (*C*) RPP14 and its dORF-encoded protein, HTD2. The *RPP14* mRNA encodes two ORFs. The canonical ORF encodes a member of the ribonuclease P (RNase P) complex (RPP14) involved in tRNAs maturation. In the 3′ UTR, a second ORF encodes a mitochondrial dehydroxylase, HTD2. HTD2 is involved in mitochondria fatty acid synthesis. (*D*) *ATXN1* is a dual coding gene. *ATXN1* mRNA encodes two proteins, ataxin and alt-ataxin. Upon entry into the nucleus, ataxin binds the transcription factor capicua (CIC) and associates with DNA at transcription sites. Ataxin nuclear localization and transcription are necessary for alt-ataxin nuclear import and its interaction with ataxin in nuclear inclusions. Ataxin is thought to shuttle between CIC complexes and RNA-binding RBM17 complexes. Polyglutamine extensions in ataxin are responsible for spinocerebellar ataxia type 1 (SCA1) and alter the dynamics of ataxin localization, thereby altering gene expression.

The field of smORFs is rapidly expanding. With the implementation of large-scale proteogenomics and ribosome profiling studies for smORF detection, their discovery is becoming less serendipitous (Ma et al. 2016; Delcourt et al. 2017; Willems et al. 2017). One of the first and most striking examples is that of the apelin (*APELA* smORF), 58 amino acids (aa), shown to bind apelin receptors (Pauli et al. 2014). Since then, even smaller apelin variants have been discovered (Huang et al. 2017). All isoforms originate from a 77-aa precursor, pre-proapelin (Fig. 2A; Lee et al. 2005; Castan-laurell et al. 2012). Apelin stimulates several metabolic pathways, such as glucose uptake, mitochondrial biogenesis, and fatty acid oxidation, while inhibiting lipolysis and insulin secretion (Boucher et al. 2005; Dray et al. 2008; O'Carroll et al. 2013; Alfarano et al. 2015; Bertrand et al. 2015). Rapidly, apelin went from an overlooked ORF in a lncRNA to a promising biomarker and therapeutic target in cardiovascular diseases, diabetes, and diabetic complications (Castan-Laurell et al. 2011, 2012; O'Carroll et al. 2013; Hu et al. 2016; Huang et al. 2018). Elevated apelinemia was found in obese patients across several studies, which was suggested to be a compensatory mechanism prior to insulin resistance (Boucher et al. 2005; Heinonen et al. 2005, 2009; Li et al. 2006; Castan-Laurell et al. 2008; Dray et al. 2008, 2010; Erdem et al. 2008; Soriguer et al. 2009; Telejko et al. 2010). Both short- and long-term apelin treatments in insulin-resistant obese mice were proven to improve insulin sensitivity (Dray et al. 2008; Castan-Laurell et al. 2011; Hu et al. 2016). *APELA* annotation has now changed from lncRNA (GRCh37) to mRNA (GRCh38), highlighting the dynamic nature of genome annotations (Delcourt et al. 2017). Other biological roles attributed to smORFs include sarcoendoplasmic reticulum calcium transport ATPase (SERCA) machinery regulation, regulation of ribosome-protein complexes, prevention of cell death, and regulation of transcription (Hashimoto et al. 2001; Galindo et al. 2007; Kondo et al. 2007; Hanyu-Nakamura et al. 2008; Escobar et al. 2010; Magny et al. 2013; Anderson et al. 2015; Pueyo et al. 2016b; D'Lima et al. 2017; Matsumoto et al. 2017a, b). Moreover, smORFs have been detected within the mitochondrial genome, encoding short circulating peptides acting in a hormone-like manner (Yen et al. 2013; Lee et al. 2016; Kim et al. 2017; Okada et al. 2017).

These multiple reports of smORFs, often encoded in lncRNAs, highlight the previously hidden coding potential of lncRNAs (Niazi and Valadkhan 2012; Slavoff et al. 2013; Ruiz-Orera et al. 2014; Ji et al. 2015). Admittedly, not all lncRNAs are misannotated, and evidence that these transcripts act as functional RNAs rather than protein coding RNAs is not to be dismissed (Guttman et al. 2013).

## Upstream ORFs: ORFs encoded in the 5′ UTR of mRNAs

Advances in large-scale ribosome profiling led to the discovery of widespread translation events outside of annotated CDS (Ingolia 2014, 2016). A large portion of these events were observed upstream of annotated CDS, in the 5′ UTR (Ingolia et al. 2009). Translation of these upstream ORFs (uORFs) was first described as a regulatory mechanism for the translation machinery. Indeed, several examples show that mutations creating or suppressing an uORF led to a decrease or increase in the downstream canonical protein expression (Cabrera-Quio et al. 2016). One of the best studied examples of protein expression regulation by uORF translation is that of the GCN4 protein (Natarajan et al. 2001). The *GCN4* transcript contains four uORFs that ensure a tightly regulated expression of the CDS, a transcription factor. GCN4 protein targets most genes required for amino acid biosynthesis (Natarajan et al. 2001). Upon starvation, translation re-initiation at the multiple uORFs is down-regulated and the GCN4 protein expression level thus rises (Hinnebusch 2005; Gunišová et al. 2016). Multiple examples of uORF-mediated regulation of protein levels have been published; however, numerous studies also highlight the biological role of uORF-encoded peptides (Lee et al. 2014; Cabrera-Quio et al. 2016; Plaza et al. 2017). In 2004, a proteomics study detected 54 novel microproteins mapped back to uORFs (Oyama et al. 2004) and 40% of identified smORF-encoded peptides (SEPs) were from uORFs in Slavoff et al. (2013). At least two of these uORF peptides were shown to be functional proteins (on *SLC35A4* and *MIEF1* transcripts), and several others are conserved and likely to be of biological importance (Vanderperre et al. 2013; Andreev et al. 2015; Ebina et al. 2015; Young and Wek 2016). The *SLC35A4* transcript was shown to be resistant to stress (sodium arsenite), and uORF-encoded alt-SLC35A4 was shown to positively regulate SLC35A4 protein translation in the context of the integrative stress response (Fig. 2B). Alt-SLC35A4 expression levels remained unchanged following sodium arsenite treatment (Andreev et al. 2015; Ma et al. 2016).

## Downstream ORFs: ORFs encoded in the 3′ UTR of mRNAs

Targeted proteomics for small peptides has also increased the detection of proteins mapped back to 3′ UTR ORFs, downstream from an annotated CDS (dORFs). Sixteen percent of the identified SEPs in Slavoff's study were from dORFs (Slavoff et al. 2013). To the best of our knowledge, only one 3′ UTR encoded protein has been functionally characterized thus far (Autio et al. 2008). HTD2 (hydroxyacyl-thioester dehydratase type 2) is localized on *RPP14* mRNA, downstream from the canonical sequence (Fig. 2C). RPP14 is a component of the ribonuclease P (RNase P) complex necessary for tRNA maturation, while HTD2 is a mitochondrial protein involved in mitochondrial fatty acid biosynthesis (Autio et al. 2008). Evolutionary analysis of RPP14 and HTD2 sequences highlight a conserved bicistronic relationship over 400 million years and thus suggest a functional link between RNA processing and mitochondrial fatty acid synthesis (Autio et al. 2008). Numerous ribosome profiling and mass spectrometry studies highlight translation events in the 3′ UTR and dORF-encoded peptide detection (Slavoff et al. 2013; Ingolia 2016). For example, the OpenProt database reports 5180 predicted dORFs detected by mass spectrometry and 535 by ribosome profiling, including 41 detected by both techniques (openprot.org). The SmProt database reports no dORFs in their mass spectrometry data set but 2389 in their ribosome profiling data set (Hao et al. 2017). The small ORFs repository (sORFs.org) reports 44,163 dORFs detected by ribosome profiling (Olexiouk et al. 2016).

## Polycistronic regions: overlapping ORFs on one mRNA

Finally, hundreds of unannotated ORFs overlapping a canonical CDS have been described as well (Vanderperre et al. 2011, 2013; Bergeron et al. 2013; Slavoff et al. 2013). These ORFs are at the same locus as an annotated CDS but encoded in an alternative reading frame and can either partially overlap the CDS or be completely nested within it. Several mammalian polycistronic mRNAs have been reported over the past decade (for review, see Karginov et al. 2017). These overlapping ORFs might be more common than previously thought, given that 30% of peptides from Slavoff's study were mapped back to overlapping ORFs (Slavoff et al. 2013). The OpenProt database reports 4916 alternative proteins from overlapping ORFs detected by mass spectrometry and 3756 by ribosome profiling, including 268 detected by both techniques (openprot.org). For example, the *ATXN1* gene, involved in spinocerebellar ataxia type 1, was identified as a dual coding gene (Fig. 2D). The canonical gene product, ataxin, is a chromatin-binding factor and is thought to have a role in RNA metabolism (Yue et al. 2001). The alternative protein product, alt-ataxin, directly interacts with ataxin and poly(A)^+^RNAs (Bergeron et al. 2013). Polyglutamine extensions in ataxin are responsible for spinocerebellar ataxia type 1 (SCA1). Normally, upon entry into the nucleus, ataxin binds the transcription factor capicua (CIC). Ataxin-CIC complexes then associate with DNA at transcription sites (Lim et al. 2008). Alt-ataxin is diffusely localized in the nucleus in the absence of ataxin, but in the presence of ataxin it readily colocalizes in nuclear inclusions (Bergeron et al. 2013). Ataxin is thought to equilibrate between CIC complexes and RNA-binding RBM17 complexes, which regulates transcription and RNA processing, notably splicing. In the case of SCA1, the polyQ extensions favor ataxin-RBM17 complexes over those with CIC, thereby competing with CIC containing complexes and altering gene transcription (Lim et al. 2008; Paulson et al. 2017).

As illustrated by this last example, proteins encoded within the same mRNA often share a functional link. Most fall into three categories: (1) a direct protein interaction, either in a complex or as a chaperone (Quelle et al. 1995; Bergeron et al. 2013); (2) a positive functional interaction (involved in the same pathway but at distinct points and expression levels) (Abramowitz et al. 2004); and (3) a negative functional interaction (two proteins involved in the same pathway, with opposite roles) (Lee et al. 2014).

## The clinical and research need for a better annotation system

This growing body of evidence for functional alternative ORFs calls attention to the need for a novel genome annotation (Yen et al. 2013; Pauli et al. 2014; Anderson et al. 2015; Lee et al. 2016; D'Lima et al. 2017; Huang et al. 2017). Indeed, the current overly restrictive definition of a gene inhibits research and clinical advances. The field is facing a vicious cycle phenomenon: Most alternative ORFs are not identified as new genetic drivers or pathological causes since they are not annotated. Yet, genome annotations, faced with the challenge of distinguishing functional ORFs from random events, do not include alternative ORFs. That is because of a lack of clinical importance and/or experimental evidence for alternative ORFs (Couso and Patraquim 2017). However, this paucity of evidence is largely due to their absence from current annotations (Cheng et al. 2011; Ladoukakis et al. 2011; Andrews and Rothnagel 2014; Saghatelian and Couso 2015; Couso and Patraquim 2017).

**Figure 3.**
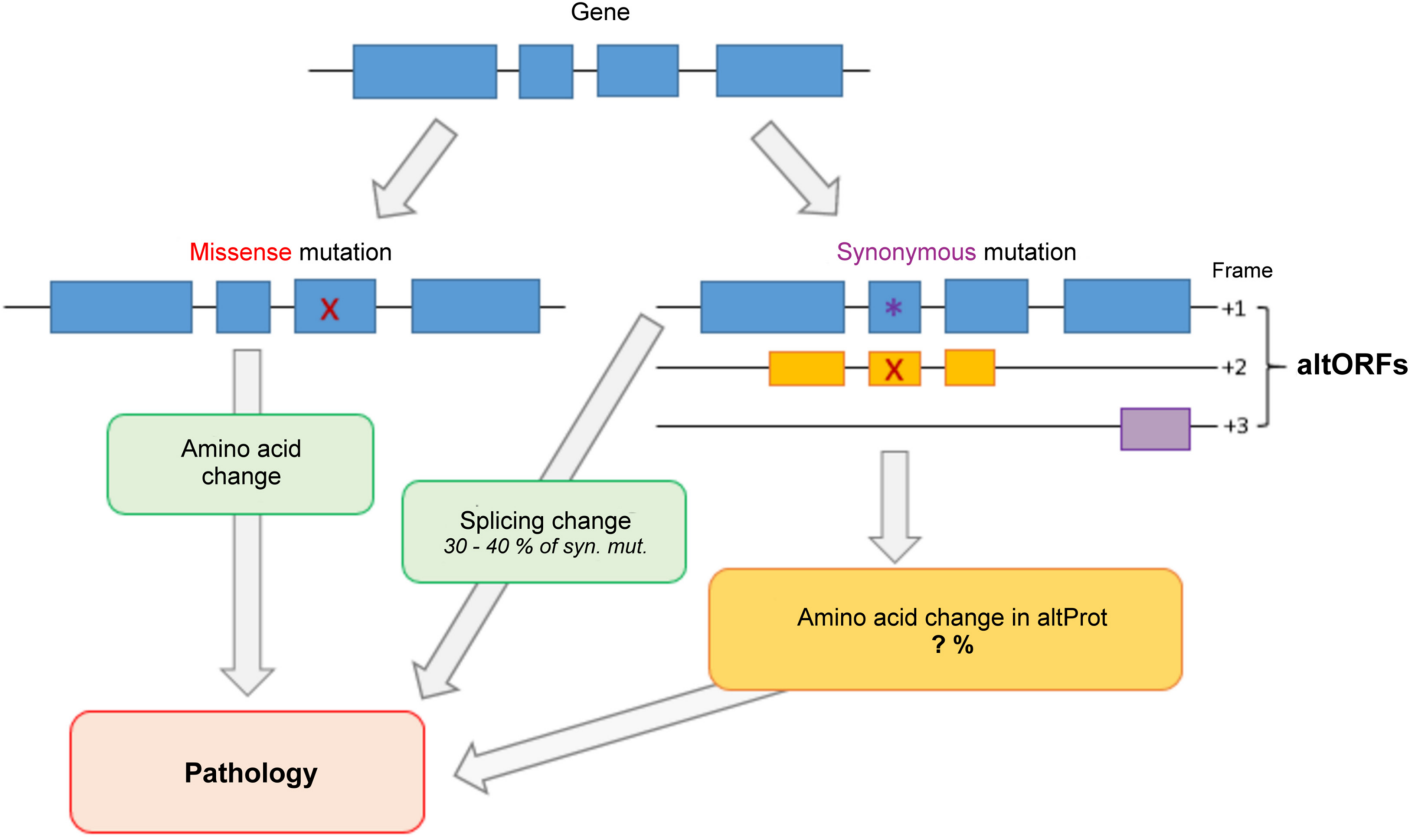
Graphical representation of ways a genetic mutation might cause pathology. Mutations from a single nucleotide variation (SNV) can result either in a missense mutation (red X) or in a synonymous mutation (purple star). Missense mutations are the most studied, as they lead to an amino acid change in the gene's annotated protein sequence. Synonymous mutations are studied mostly for their likelihood to alter splicing sites (about 30% of cases). However, a synonymous mutation in a gene's annotated protein sequence (in blue) might cause an amino acid change in a protein encoded in an alternative open reading frame (altORF/altProt—in yellow). These altered proteins might be a yet unexplored mechanism by which a SNV is pathological.

Genome annotations are the linchpin to today's research and clinical screening, and the practical impact of their incompleteness is thus substantial. With the development of time-efficient, reproducible, and cost effective Next Generation Sequencing (NGS), the amount of genome and exome sequencing data is no longer a major limit for today's clinical screening and research (Boycott et al. 2013; Goodwin et al. 2016). Indeed, an increasing number of genes have been related to pathological germline and somatic mutations since the use of NGS (Vogelstein et al. 2013; Amberger et al. 2015). Yet, only about 35% of exome sequencing tests result in the identification of a likely pathological mutation (Ku et al. 2016). This is partly due to the current recommendations from the American College of Medical Genetics and Genomics (ACMG), which considers likely pathological mutations from a uni-coding dogma point of view (Richards et al. 2015). The uni-coding dogma establishes that one gene encodes one protein and its splicing-derived isoforms. Thus, single nucleotide variants (SNVs) resulting in missense mutations are considered, but those resulting in synonymous mutations are often ignored unless they alter a splicing site or have a known functional consequence (Richards et al. 2015). Admittedly, a challenge coming with such a wealth of data is to distinguish single nucleotide polymorphisms (SNPs), or passenger mutations in cancer, from pathological SNVs (Makrythanasis and Antonarakis 2013; Vogelstein et al. 2013; Tokheim et al. 2016). So far, the response to this dilemma has been to use more stringent criteria for linking SNPs to pathologies, and synonymous mutations are often discarded and regarded as silent mutations under a uni-coding dogma (Nielsen et al. 2011, 2012; Olson et al. 2015). However, a synonymous mutation in one reading frame may be a missense in another and could thereby represent a pathological alteration for an alternative ORF (Fig. 3). In fact, synonymous mutations have been described in several pathologies, from cancer to neurological disorders (Sauna and Kimchi-Sarfaty 2011; Supek et al. 2014; Fahraeus et al. 2016; Li et al. 2016b; Waters et al. 2016; Austin et al. 2017; Batista et al. 2017; Soussi et al. 2017). The mechanisms put forward to explain a pathological outcome from a silent mutation mostly revolve around the stability of the mRNA, its splicing, or the protein folding (Fahraeus et al. 2016). Yet, even these mechanisms only explain about a third of pathological synonymous SNVs (Supek et al. 2014).

Here, we suggest that alternative proteins might explain these additional pathological SNVs (Fig. 3). A silent mutation in an annotated protein might alter a second protein encoded in an alternative ORF in the same mRNA. The underlying pathological cause could thus be an amino acid change in that second protein, previously “hidden” because it is not annotated in genome databases. As an example, we explored Supek's study from 2014 (Table 1; Supek et al. 2014). In that study, synonymous mutations were identified as drivers in human cancers. For each gene found enriched in synonymous mutations in Supek's study, we gathered synonymous mutations coordinates from the The Cancer Genome Atlas (TCGA) database (The Cancer Genome Atlas Research Network 2013, 2016; Supek et al. 2014; Favazza et al. 2017). In the first data set, 25 oncogenes were found enriched in synonymous mutations in a tissue-specific manner. Synonymous SNVs coordinates from the TCGA database for each specific tissue were checked against genomic coordinates of predicted alternative ORFs (Table 1; openprot.org). Sixty-four percent of genes displayed at least one “synonymous” SNV altering the amino acid sequence of at least one predicted alternative protein. We consider here any type of alterations, be it missense, nonsense, frameshift, or point mutations. Of all listed synonymous SNVs within these 25 genes, in the specific tissues, 29.6% fell within a predicted alternative protein. Out of these, 7% have been detected in ribosome profiling and reanalysis of large-scale mass spectrometry studies (openprot.org). The majority of these predicted alternative proteins are small proteins with a median length of 48 aa.

**Table 1.**
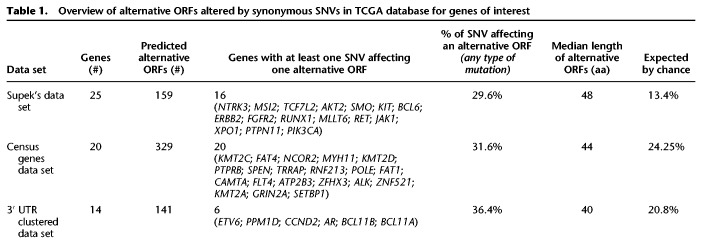
Overview of alternative ORFs altered by synonymous SNVs in TCGA database for genes of interest

In the second data set, we gathered the top 20 Census genes harboring the most synonymous SNVs in the TCGA database and repeated the analysis (Futreal et al. 2004). All genes displayed at least one synonymous SNV altering at least one predicted alternative protein. About 30% of all listed synonymous SNVs within these 20 Census genes fell within a predicted alternative protein (Table 1). Out of these, 7.3% have been detected in re-analysis of large-scale mass spectrometry studies (openprot.org). The majority of these predicted alternative proteins are small proteins with a median length of 44 aa.

Finally, at the end of their publication, Supek et al. (2014) provided a list of clustered SNVs in the 3′ UTR of 14 different genes associated with human cancers. We checked this list of SNVs coordinates against that of alternative proteins within these genes. Of these mutations, 31.6% fell within a predicted alternative protein (Table 1). Again, the majority of these predicted alternative proteins are small proteins with a median length of 40 aa.

All of these percentages were higher than expected by chance (Table 1).

The absence of most of the alternative ORFs from genome annotations prevents us from identifying novel genetic drivers. As of today, only about 62% of Mendelian phenotypes have a known molecular basis, consistent with the hypothesis that at least some of these phenotypes result from defective alternative proteins (Amberger et al. 2015). A striking example is provided by one of the first discovered smORFs, apelin. Since its change in annotation from lncRNA to mRNA following its incidental discovery and functional characterization, genomic studies have identified polymorphisms linked to cardiovascular diseases and obesity risks (Zhao et al. 2010; Liao et al. 2011; Jin et al. 2012; Sentinelli et al. 2016). Finally, many published studies may need to be re-interpreted in light of the existence of more than one CDS per mRNA, and future overexpression and knockdown experiments will become technically more complex. For example, transfection of a CDS might actually result in the overexpression of two proteins, which are often functionally related (Klemke et al. 2001; Bergeron et al. 2013; Delcourt et al. 2017). This also means that the knockdown or knockout of genes could result in the absence of two or more proteins rather than one.

## Proposition of a novel annotation framework

It is difficult to come up with a genome annotation pipeline that is both accurate and exhaustive, yet the need is evident. Different strategies have been adopted over the past years, which essentially regroup two goals: (1) to identify transcript structure (e.g., intron vs. exon); and (2) to identify the functional potential (e.g., contains a CDS) (Pruitt et al. 2009; Harrow et al. 2012; Aken et al. 2016; Mudge and Harrow 2016). These pipelines, however, invoke a uni-coding presumption. ORF-prediction algorithms apply the criteria of a single CDS per transcript, and a minimum length of 100 codons, unless the sequence bears high similarity to known proteins or domains (Furuno et al. 2003; Pruitt et al. 2012; Aken et al. 2016). As a result, the foreseen increase in smORF count in Swiss-Prot falls short, with an increase from 3.1% in 2009 to 3.3% in 2017 (Southan 2017). This means that despite the large number of smORF and alternative ORF discoveries, only a limited number make it through to genome annotation (Southan 2017). The current genome annotation system has been blamed for simplifying a transcript's definition, not taking into account their potential to hold multiple functional features (for review, see Mudge and Harrow 2016).

**Figure 4.**
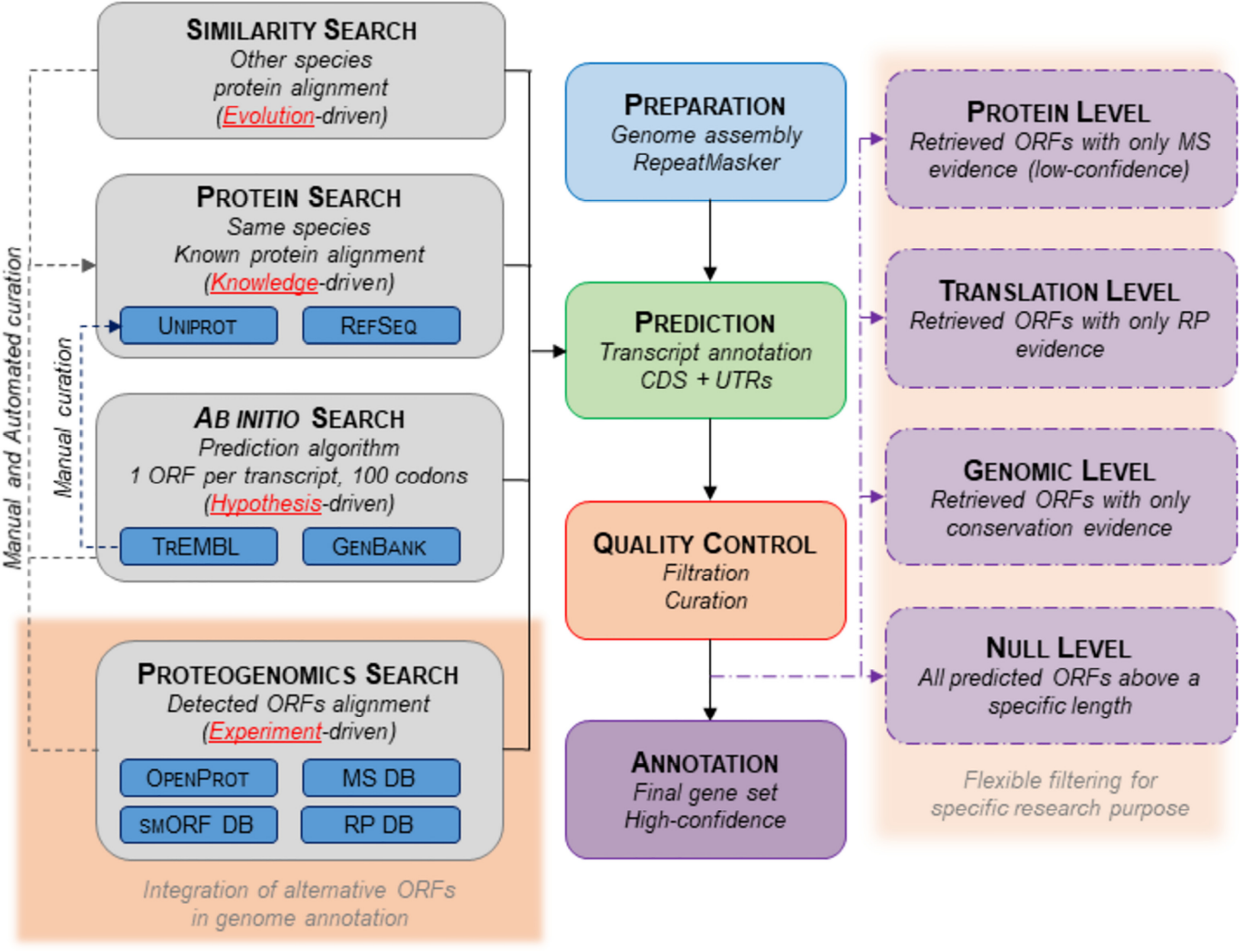
Proposed novel genome annotation framework. Current genome annotations’ pipelines have four main steps: Preparation, Prediction, Quality Control, and Annotation. The Prediction step aims to annotate transcripts (exons, introns) and CDSs (with flanking UTRs). It mostly relies on three methods: a search by homology (different species known proteins are aligned to the genome assembly), a search by prior knowledge (same species known proteins are aligned to the genome assembly), and a search ab initio (prediction of ORFs by algorithms). Here, we suggest adding an experiment-driven search and including alternative ORFs with experimental detection. The output could also be flexible to fit different experimental purposes. The pipeline steps highlighted in red correspond to the suggested implementation.

Here, we propose a framework for the incorporation of alternative ORFs into current genome annotations. With minimal modifications to the existing annotation pipelines (GENCODE, Ensembl, or NCBI for the human genome), alternative ORFs could be included (Harrow et al. 2012; Pruitt et al. 2012; Aken et al. 2016). As shown in Figure 4, most pipelines annotate ORFs and subsequent protein products from ab initio ORF prediction or sequence alignment with known proteins (from the UniProt or RefSeq databases) (Keller et al. 2011; The UniProt Consortium 2014). ORF prediction mostly relies on ORF size, codon usage, and the nonsynonymous to synonymous mutation ratio (Pruitt et al. 2009; Keller et al. 2011; Mudge and Harrow 2016). This means that current genome annotations are shaped by evolution-, prior knowledge-, and hypothesis-driven data. As proposed in Wright et al. (2016) for the emerging field of proteogenomics, protein sequences from alternative ORFs, reported in databases such as OpenProt (openprot.org), sORFs (Olexiouk et al. 2016), or SmProt (Hao et al. 2017), with detection evidence by ribosome profiling or mass spectrometry, could be downloaded for genome annotation (Fig. 4). Such an annotation pipeline would prevent some of today's pitfalls, abolishing the unique CDS presumption and empowering experimental data as well as conservation signatures (Mudge and Harrow 2016; Southan 2017). This would add a layer of experiment-driven data to genome annotation pipelines.

One of the biggest challenges for genome annotation will be to distinguish random ORFs from functional ones. Random ORFs are ORFs that could arise through evolutionary noise, e.g., a mutation causing a start codon to appear randomly within a transcript. Random ORFs could potentially be translated and thus be a source of translational noise but would not usually yield a functional detectable peptide (Brar and Weissman 2015). Purifying selection is expected to weed out detrimental random ORFs relatively quickly for dominant traits but more slowly for neutral random ORFs. It is not known what percentage of alternative ORFs predicted based on transcript sequences are random. Obviously, we would like to exclude random ORFs from annotations. However, the better we exclude random ORFs, the more functional ORFs will also be excluded, analogous to problems of true and false positives in medical diagnostics. The short length of alternative ORF sequences means that, for statistical reasons, either the false positive or false negative rate will be higher than for longer sequences.

While we believe that current annotation methods are too restrictive, there is also a real interest in avoiding false positives. The relative balance between inclusivity and exclusivity (sensitivity and specificity) will depend strongly on the experimental context and the questions being asked. To deal with these complexities, we propose a solution where annotations include filters that allow researchers to adjust the levels and types of evidence for annotated proteins. Evidence can be inferred from large-scale detection methods, either at the DNA (conservation signatures), the translation (ribosome profiling), or the protein level (mass spectrometry). And even though there is no perfect detection method for alternative proteins, one should be cognizant of each technique's strengths and pitfalls and strive to use and adapt them to better detect the entire proteomic landscape of a cell or tissue (Boekhorst et al. 2011; Aspden et al. 2014; Calviello et al. 2016; Hellens et al. 2016; Ma et al. 2016; Pueyo et al. 2016a; Delcourt et al. 2017; Hsu and Benfey 2017; Willems et al. 2017).

### Evidence at the genomic level

**Figure 5.**
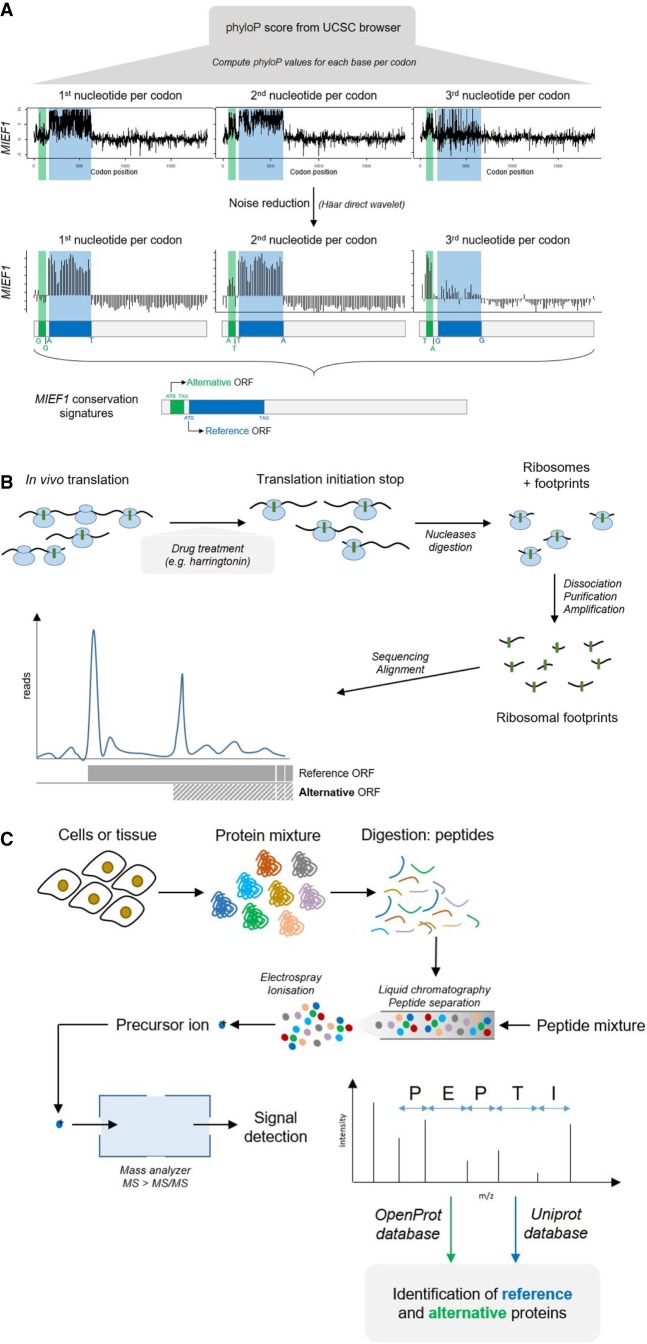
Large-scale detection methods for alternative proteins detection. (*A*) Conservation signatures of proteins encoded in different reading frame from the same mRNA. PhyloP scores can be computed from the UCSC Genome Browser, and noise filtration (by Haar direct wavelet) allows for the identification of distinct purifying selection signals in each reading frames. Here, the example of the dual coding *MIEF1* gene is represented and corroborates data from mass spectrometry and ribosome profiling with the detection of an alternative ORF upstream of the canonical CDS (reference ORF). (*B*) Schematic representation of the ribosome profiling technique. This technique allows for detection of ribosomal footprints, and subsequent mapping on the genome yields a map of translation events throughout. Translation initiation at alternative ORFs can then be detected. (*C*) Schematic representation of the mass spectrometry technique. The search space bears crucial consequences on peptide identification. Here, we represent the strategy used by the OpenProt database that re-analyzed published mass spectrometry studies adding their predicted alternative ORFs to the scope of possibilities.

An indirect but potentially powerful piece of evidence of a protein's expression is its conservation signature. Conservation signatures are already used to distinguish functional ORFs in current ORF prediction algorithms (Mudge and Harrow 2016). Functional proteins are expected to be under purifying selection and the ratio of nonsynonymous to synonymous mutations highlights protein-coding sequences (Hughes 1999; Pál et al. 2006). The first and second nucleotide of a codon experience stronger selection than the third because of the genetic code's redundancy (Pollard et al. 2010; Samandi et al. 2017). This selection periodicity can allow for detection of conservation signatures in each of the reading frames (Fig. 5A). The phyloP score (a measure of probability to be under purifying selection) can be computed for every third base giving a triplet signal (three graphs corresponding, respectively, to the first, second, and third nucleotide for each codon) (Cooper et al. 2005; Samandi et al. 2017). After noise reduction, we can detect independent purifying selection signals in each of the reading frames, e.g., for the dual coding *MIEF1* gene (Fig. 5A). This method allows for annotation of genetic loci under purifying selection, but it relies on a good signal-to-noise ratio (and id facto on genome annotations for other species). However, this ratio may be biased by the phyloP score itself. Indeed, the phyloP score first evaluates the rate of neutral evolution for one locus based on empirical values of substitution rates, but these have been defined under a uni-coding gene presumption (Cooper et al. 2005). Moreover, some alternative ORFs could be the result of a more recent evolution and still be in a phase of adaptive selection (Ruiz-Pesini et al. 2004; Evans et al. 2014; McLysaght and Hurst 2016). Other measures of phylogenetic evolution can be used, such as PhyloCSF or CPC (Coding Potential Calculator), and Bazzini et al. combined evolutionary methods (PhyloCSF) to ribosome profiling (Kong et al. 2007; Lin et al. 2011; Bazzini et al. 2014). PhyloCSF uses the widely implemented phylogenetic analyses by maximum likelihood (Yang 1994, 2007), but it still relies on previously empirically determined matrices of codons’ transition rates (ECMs; Empirical Codon Models). These ECMs were defined under a uni-coding gene presumption and could thereby bias the PhyloCSF score (Kosiol and Goldman 2005; Kosiol et al. 2007; Lin et al. 2011). The CPC score is designed to measure the coding potential of a transcript and uses machine-learning algorithms. However, the true nature (coding or noncoding) of the transcripts used in the training data set would be a critical element to the CPC's performance (Kong et al. 2007; Halevy et al. 2009). Conservation signatures may improve in the near future as new algorithms take into account the multicoding potential of mature mRNAs.

### Evidence at the translational level

Ribosome profiling is a technique that measures ribosomal occupancy and initiation in vivo using deep sequencing of ribosome-protected mRNA fragments. First described by Steitz, ribosome profiling was recently adapted by Ingolia to make use of NGS techniques and is now a widely used technique to describe the full coding potential of a genome (Steitz 1969; Ingolia et al. 2009, 2012; Ingolia 2014). In brief, the idea is to sequence ribosomal footprints, given that each ribosome encloses about 30 nucleotides when translating and thus protects them from nuclease digestion (Fig. 5B). These footprints can be amplified, sequenced, and mapped on the genome, thus identifying in vivo translation events (Ingolia et al. 2012). Ribosome profiling techniques have also been adapted to specifically isolate initiating ribosomes. Using drugs that stall the first step of elongation (harringtonine, lactimidomycin with puromycin), all initiation sites can be mapped on the genome (Ingolia et al. 2011; Ingolia 2016). However, the accuracy of ribosome profiling depends on fragment mapping on the genome, and since fragments are short, this creates a risk of multimapping (multiple match) and a bias against repetitive regions. There is also evidence that some genuine ribosome profiling identifications do not lead to the translation of functional proteins but rather are regulatory ribosome-RNA interactions (Ingolia 2016; Raj et al. 2016). Nonetheless, ribosome profiling offers a translation overview of the genome that is evolution-free, meaning that nonconserved or de novo translated ORFs would still be identified. There is also a dogma that function implies conservation, and accordingly, the possibility to identify nonconserved yet functional proteins arouses strong opinions (The ENCODE Project Consortium 2012; Graur et al. 2013; Han et al. 2014). Available online tools for visualization of ribosome profiling data are listed in Table 2.

**Table 2.**
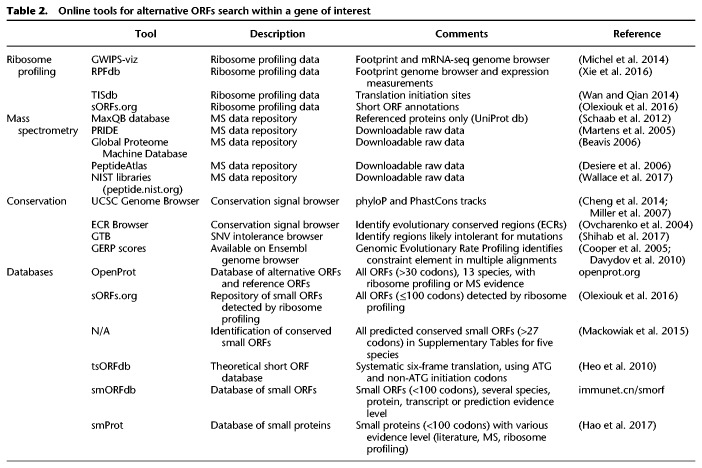
Online tools for alternative ORFs search within a gene of interest

### Evidence at the protein level

Mass spectrometry (MS)-based proteomics has emerged as the gold standard technique to assess the protein landscape of a cell or tissue and thus can offer additional evidence beyond ribosome profiling (Aebersold and Mann 2003, 2016; Vogel and Marcotte 2012; Huttlin et al. 2015). Cells or tissue lysates are digested to peptides, subsequently identified by mass spectrometry (Fig. 5C). However, the scope of the search space has a substantial impact on the proportion of peptide identification (Aebersold and Mann 2003, 2016). Peptide identification relies on matching mass spectra to predicted peptides from CDS (e.g., from UniProt database). If the database does not contain the relevant peptide, the associated protein will never be identified since it is not included in the scope of possibilities (Samandi et al. 2017). As of today, <50% of all MS/MS spectra from a proteomics experiment are matched with high confidence (Heo et al. 2010; Chick et al. 2015). These unassigned peptides can correspond to peptide modifications or to proteins not in the database (Heo et al. 2010). In the recently developed proteogenomics approaches, addition of more inclusive databases to the search space allows for the discovery of novel proteins thus far undetected (Oyama et al. 2007; Saghatelian and Couso 2015; Samandi et al. 2017; openprot.org). Yet, not all proteins produce peptides detectable by mass spectrometry, owing to their subcellular localization, chemistry, and/or size. This is partly why false-discovery rates in proteomics experiments can be difficult to evaluate (Nesvizhskii 2014). Alternative ORFs are smaller than canonical CDS, with a median length of 45 aa (Samandi et al. 2017), and mass spectrometry detection of small and low-abundance proteins is challenging (Nesvizhskii 2014; Aebersold and Mann 2016). Identification of any protein by mass spectrometry relies heavily on good quality spectra, but this is particularly true for alternative proteins, as most smaller proteins will produce fewer peptides upon enzymatic digestion (Ma et al. 2016). There could also be cases where a protein might not produce any peptides from trypsin digestion (most used enzyme) or might produce highly hydrophilic peptides, rendering its identification by proteomics challenging (Young et al. 2017). Nonetheless, specific proteomics protocols to better detect small proteins are emerging and raise hopes for the future of proteogenomics in genome annotation (Ma et al. 2016). Table 2 contains a list of online tools available for alternative ORF mass spectrometry identification.

### Available online resources

Several online tools either allow for raw data enquiry or provide a list of all alternative ORFs with corresponding evidence of expression for several species (see Table 2). Moreover, some tools also predict the translation of alternative ORFs, such as SPECtre, RiboTaper, ORF-RATER and PROTEOFORMER (Crappé et al. 2015; Fields et al. 2015; Calviello et al. 2016; Chun et al. 2016), based on integration of ribosome profiling data. PROTEOFORMER and RiboTaper combine ribosome profiling data with an implemented construction of protein sequences from thus detected ORFs. Thereby, they build a database that can be used for proteomics without leading to a large increase in the proteomic search space (Jeong et al. 2012; Crappé et al. 2015; Guthals et al. 2015). Some databases of alternative ORFs also offer a freely downloadable FASTA file for proteomics experiments (openprot.org).

## On the importance of filtration and curation

There is an undeniable close relationship between the quality of a genome annotation and experimental and clinical results. That is why all genome annotation pipelines include a step of database filtration and curation (Pruitt et al. 2012; The UniProt Consortium 2014; Aken et al. 2016; Tatusova et al. 2016). Often, the first step (Prediction on Fig. 4) emphasizes sensitivity over specificity. However, because false positives could burden variant-calling workflows, putative functional annotations are removed at the filtration and curation steps (Quality Control on Fig. 4; Koonin and Galperin 2003; Mudge and Harrow 2016). However, as discussed earlier, genome specificity needs might differ based on the experimental purpose (variant calling, novel protein identification, etc.). The RefSeq database offers some more putative functional annotation (XM_, XP_ annotations), and Ensembl reports to some extent less supported transcripts’ annotations (Pruitt et al. 2012; Aken et al. 2016). Yet, these still rely on overly restrictive criteria (one CDS per transcript, longer than 100 codons) (Chung et al. 2007; Galindo et al. 2007; Saghatelian and Couso 2015; Pueyo et al. 2016a; Couso and Patraquim 2017). While adapting the framework of genome annotations to consider alternative ORFs will more likely yield significant advances, the need for a more flexible annotation for various purposes could be addressed (Fig. 4). The different levels of confidence suggested in Figure 4 are based on evidence levels discussed earlier: conservation, ribosome profiling, mass spectrometry, or none of the aforementioned.

### The complexity behind the data sets

In the suggested annotation pipeline, we emphasize experiment-driven annotations. In that aspect, the pipeline would rely on the data quality of the databases used (OpenProt, sORF, SmProt, etc.) (Fig. 4). It is important to note that although implementation of identifications from these databases would be straightforward, the quality control of the data might not be. Indeed, as mentioned earlier, the various databases present discrepancies in numbers of identifications. They uphold different definitions of alternative ORFs, but they also enforce different identification pipelines. All methods suggested here—ribosome profiling, proteogenomics, and conservation signatures—are noisy and require adequate filtering and thresholding to minimize the risk of false positives (Guthals et al. 2015; Aebersold and Mann 2016; Ingolia 2016; Calviello and Ohler 2017; Wallace et al. 2017). We would recommend databases using raw data and an adequate pipeline of identification. For example, a two-stage FDR pipeline could be used for mass spectrometry, in order to minimize the impact of the increased search space (Woo et al. 2014, 2015; Pauli et al. 2015). The use of additional algorithms in order to control for misidentification of post-translational modifications would be encouraged (Kong et al. 2017). In ribosome profiling, multimapping should be filtered out, keeping only unique mappings, with an appropriate sequencing depth threshold (Calviello and Ohler 2017). Moreover, elongating reads and RNA-seq data will strengthen the observations (Calviello and Ohler 2017).

### Is ORF length an appropriate filter?

The rationale behind the minimum ORF length of 100 codons is to avoid polluting annotations with random events (Pruitt et al. 2009). Yet, it is clear it also leads to numerous false negatives, i.e., functional ORFs shorter than 100 codons excluded from annotations (Andrews and Rothnagel 2014; Ma et al. 2014; Pauli et al. 2014; Couso and Patraquim 2017). Notwithstanding, we could also question the arbitrary cut-off taken by groups studying alternative ORFs. For instance, the smORF community only reports ORFs shorter than 100 codons, but they would then miss all longer alternative ORFs (Cabrera-Quio et al. 2016; Hellens et al. 2016). The OpenProt team does not limit itself to alternative ORFs shorter than 100 codons, but it still uses an arbitrary minimal cut-off of 30 codons (openprot.org). This 30-codon cut-off allows for prediction of multiple alternative ORFs (361,173 unique alternative ORFs predicted in the human genome) without overcrowding the search space for proteomics experiments (Jeong et al. 2012; Nesvizhskii 2014; Guthals et al. 2015). However, examples of smORFs shorter than 30 codons have been published, and it questions the adequacy of an ORF length threshold (Yosten et al. 2016). The aforementioned genome annotation framework would still rely on some arbitrary ORF length cut-offs. Users should be aware of it and, because accumulation of random events with a lower cut-off is a statistical reality, we would recommend using the “Null Level” data set only for bioinformatics studies (Fig. 4).

### Accumulation of clinical reports as an evidence level?

The causal link from the quality of genome annotations to variant-calling misinterpretation is evident; hence, most putative annotations are removed to limit clinical false positives. Thinking about it backward, pathological family-specific variants clustered on genetic loci are a valuable yet overlooked resource. For example, in the case where no “likely pathological” variant is determined (about 65% of cases), a new variant-calling file could be generated using a less stringently filtered data set (for example, using the “Protein Level,” “Translation level,” or “Conservation level”) (Fig. 4). Thereby, mutations altering alternative proteins could be retrieved. This could generate a positive feedback loop instead of the current vicious cycle phenomenon. Likely pathological mutations, especially in the case of severe or pediatric Mendelian phenotypes, could represent a source of functional evidence (same loci, several individuals, and same family). Alternative ORFs with clinical evidence could then be annotated in the next genome annotation release.

## Foreseen consequences of implementing alternative ORFs in genome annotations

**Figure 6.**
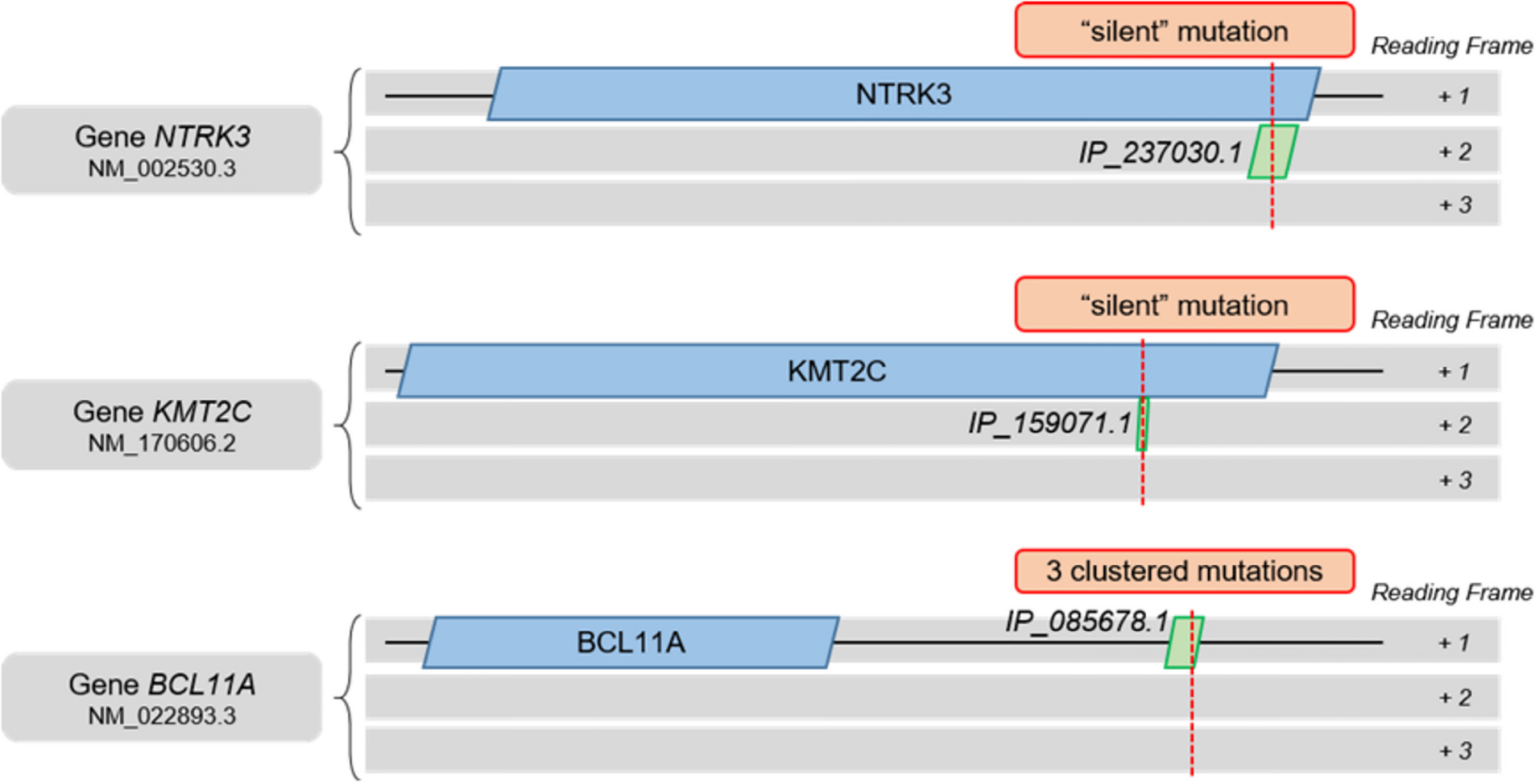
Graphical representation of alternative ORFs affected by “silent” and clustered 3′ UTR SNVs in *NTRK3*, *KMT2C*, and *BCL11A* genes. Length proportions between the full mRNA, the canonical CDS, and the alternative ORF are respected. The SNV position is represented by a red dotted line. The RefSeq transcript accession number (NM_) and the alternative ORF OpenProt accession number (IP_) are indicated.

In Supek's study on cancer-driver silent mutations (Table 1), genes containing potential alternative proteins affected by so-called “silent” mutations were identified earlier (Supek et al. 2014). Considering three genes (the top mutated for each of the three data sets), all of them present at least one alternative protein affected by such “silent” mutation or clustered mutations in the 3′ UTR (Fig. 6). These alternative proteins from the *NTRK3* and *KMT2C* genes were predicted in the OpenProt database and subsequently detected in at least one published mass spectrometry experiment re-analyzed with the OpenProt pipeline (openprot.org; Hein et al. 2015; Hurwitz et al. 2016). Thus, here are three new potential genetic drivers of human cancer.

These examples show how our current genome annotation approaches may have hidden functional proteins with pathological importance. The examples from Supek's study echo reports of human pathologies from disrupted or inserted uORFs, and studies of cellular consequences of smORF-encoded peptide disruption (Barbosa et al. 2013; Supek et al. 2014; Couso and Patraquim 2017; Plaza et al. 2017). The current body of evidence for functional alternative ORFs is but a small peek at the potential for future discoveries when their implementation in genome annotation will render identification less serendipitous. Identification of alternative ORFs will then increase, and with it, the pace of research in physiological and pathological pathways. As for the clinical side, the *APELA* gene annotation example highlights the foreseen gain. Alternative ORFs are an as-yet unexplored reservoir of genetic drivers, pathological causes, therapeutic targets, and/or biomarkers. The cooperation between fundamental and clinical research to implement and improve alternative ORFs annotation in the genome is pivotal, and it could well advance the pace of research and genomic knowledge.

Eventually, perhaps the best argument for incorporating alternative ORFs into genome annotation is to look at what might happen if we maintain the status quo. As of today, there is a dichotomy between genome annotations and experimental evidence. This gap will deepen, pulling apart genomics and proteomics. Currently, the emphasis is put on conservation signatures above all, and experimental evidence of a novel protein will not be considered if it is not followed up by a functional characterization. This means that current genome annotations provide a conceptual framework for research and medicine that is incomplete. One could question providing only partial information to the scientific community and ultimately to patients when a more exhaustive framework could be implemented. It would certainly be questionable to pollute it with random ORFs annotations. That is why we have proposed a strategy to annotate specific ORFs, with experimental evidence, rather than opening the floodgates to all alternative ORFs. Annotation censoring of alternative ORFs would likely hamper progress in alternative proteome investigations (detection, structure, and function) but also in understanding the relationship between genotype and phenotype.

## Conclusions

Current genome annotations are the linchpin to and profoundly mold today's research and genetic medicine. However, by assuming one mature RNA encodes only one protein, these annotations are incomplete. The number of functional alternative ORFs within an mRNA or a lncRNA is rapidly increasing, yet a systemic incorporation of these novel proteins into genome annotations is awaited. Hence, we need a better annotation system that can regroup the whole of transcriptomic and proteomic information contained within a gene, as suggested in Figure 4. We foresee that the implementation of such a framework would help bring attention to alternative ORFs and their potential involvement in cellular functions, pathways, and/or pathological phenotypes. Although this review focused on human genome annotations, the observations are valid for all species. Claude Bernard wrote, “It is what we know already that often prevents us from learning.” The evidence for alternative ORF translation and function is accumulating. We need to unlearn our misconception of the gene, accepting its polycistronic nature, to strive for a better understanding of the genomic complexity underlying physiological and pathological mechanisms.
